# AKL-ABC: An Automatic Approximate Bayesian Computation Approach Based on Kernel Learning

**DOI:** 10.3390/e21100932

**Published:** 2019-09-24

**Authors:** Wilson González-Vanegas, Andrés Álvarez-Meza, José Hernández-Muriel, Álvaro Orozco-Gutiérrez

**Affiliations:** 1 Automatics Research Group, Universidad Tecnológica de Pereira, Pereira 660003, Colombia; j.hernandez12@utp.edu.co (J.H.-M.); aaog@utp.edu.co (Á.O.-G.); 2 Signal Processing and Recognition Group, Universidad Nacional de Colombia, Manizales 170003, Colombia; amalvarezme@unal.edu.co

**Keywords:** approximate Bayesian computation, graph theory, kernel learning, non-linear dynamic system, statistical inference

## Abstract

Bayesian statistical inference under unknown or hard to asses likelihood functions is a very challenging task. Currently, approximate Bayesian computation (ABC) techniques have emerged as a widely used set of likelihood-free methods. A vast number of ABC-based approaches have appeared in the literature; however, they all share a hard dependence on free parameters selection, demanding expensive tuning procedures. In this paper, we introduce an automatic kernel learning-based ABC approach, termed AKL-ABC, to automatically compute posterior estimations from a weighting-based inference. To reach this goal, we propose a kernel learning stage to code similarities between simulation and parameter spaces using a centered kernel alignment (CKA) that is automated via an Information theoretic learning approach. Besides, a local neighborhood selection (LNS) algorithm is used to highlight local dependencies over simulations relying on graph theory. Attained results on synthetic and real-world datasets show our approach is a quite competitive method compared to other non-automatic state-of-the-art ABC techniques.

## 1. Introduction

Statistical inference aims to infer a set of model parameters using measured data that comes from a system under a particular scenario [1]. Usually, this kind of task is defiance due to noise present in data after the measurement stage [2]. In this sense, two main general approaches are commonly used in the state-of-the-art to proceed with the inference [3]: (i) The frequentist approach, where inference should give right answers in repeated use under an unconditional perspective that tends to focus more on analysis than on methods (e.g., convergence rates, consistency) [4]; in a frequentist approach, procedures (rule decisions) can come from anywhere, so they do not have to be explicitly derived from a probability model. (ii) The Bayesian approach, where inferences should be made conditioned on the current data under an expert-based perspective that, from prior information about the studied phenomenon, determines a probability density function for the model parameters via the Bayes’ theorem [5]; such a density, known as posterior, not only allows model checking and validation, predictive inference, and decision making but also it can tackle point and interval estimation [6].

Bayesian statistical inference tasks require to compute the likelihood function, which states how likely particular values of some statistical parameters are for a given set of observed data [1]. These approaches leverage the inclusion of a priori knowledge about the studied phenomenon into the posterior distribution. Indeed, straightforward models gather an analytic expression for the likelihood function facilitating the evidence assessment; then, the posterior can be precisely computed. Notwithstanding, for complex models, such as those that assemble high non-linearities or stochastic behavior, the model complexity means that there is no analytical formula for the likelihood function or that it is computationally intractable and can not be evaluated in any practical amount of time, standing for a really challenging scenario to perform statistical inference using Bayesian techniques [7].

Approximate Bayesian computation (ABC) emerged like a free-likelihood method to deal with the intractability mentioned above. It was originally introduced as a solution for performing statistical inference in the field of molecular biology. The first ABC algorithm was proposed to study the demographic history of the Y chromosome [8]. However, the use of ABC techniques has influenced several research areas like systems biology [9], climate analysis [10], ecological modeling [11], nuclear imaging [12], and astronomy [13], just to mention some of them. Fundamentally, an ABC method assesses an auxiliary model with different parameter values drawn from a prior distribution to calculate simulations that are compared to the observed data [14,15]. Mainly, in the face of a large number of features and observations, different authors use statistical parameters to summarize and characterize the data [16,17,18,19]. However, the selection of proper and sufficient summary statistics could be difficult for complex models. This fact has led to the need to explore alternative approaches that rely on kernel functions to embed and compare distributions into a reproducing kernel hilbert space (RKHS) [20,21]. Nonetheless, the techniques mentioned above require the estimation of different parameters related to the similarity computation among simulations to approximate the posterior. Then, expensive tuning procedures such as grid search and cross-validation are carried out. Moreover, the user requires a vast knowledge concerning the ABC algorithm and the studied data to properly tune the free parameters, yielding to a high influence in the posterior approximation quality.

In this paper, we introduce an automatic ABC algorithm, termed automatic kernel learning ABC (AKL-ABC), which comprises a kernel learning stage based on a centered kernel alignment (CKA) technique to assess the matching between similarities defined over parameter and simulation spaces in ABC [22]. This paper is an extension of our proposal presented in [23]. Namely, here we provide a series of improvements and contributions:

- We propose a novel automatic ABC approach for computing posterior estimates avoiding any tuning procedure of free parameters. In detail, a Mahalanobis distance is optimized through a CKA-based algorithm to code the simulation and parameter space matching and an information theoretic learning (ITL)-based method to learn the kernel bandwidth. Furthermore, a graph representation is carried out to highlight local dependencies utilizing a local neighborhood selection (LNS).
- The mathematical models regarding AKL-ABC are described and enhanced (including the CKA and LNS coupling with ABC through kernel machines and graph theory).
- The experiments are expanded and explained in detail considering well-known challenging databases.
- A free parameter analysis is provided to show the performance of our AKL-ABC as an automatic approximate inference method.

Achieved results on synthetic and real-world inference problems demonstrate that our AKL-ABC is robust to substantial changes in data dynamics. Namely, the experimental results show that our automatic extension of ABC is competitive with other state-of-the-art works, and has a significant advantage concerning the automatic selection of free parameters. Additionally, a MATLAB implementation of our approach is publicly released.

The remainder of this paper is organized as follows: Section 2 presents the related work. Section 3 introduces the materials and methods and provides the mathematical foundations behind our proposal. Section 4 describes the experimental setup, and the obtained results are discussed in  Section 5. Finally, the conclusions are outlined in Section 6.

## 2. Related Work

Recent progress in ABC-based inference has incorporated different research areas into the principal ABC framework to accomplish more accurate posterior estimations. While several ABC methods can be found across the literature, three main groups can be highlighted according to their main features [24]: Summary statistics-based approaches, weighting-based techniques, and regression-adjustment methods.

### 2.1. Summary Statistics-Based Approaches

The selection of proper and sufficient summary statistics is a crucial issue in ABC [25]. Wood [18] introduced a synthetic likelihood modeled as a multivariate normal whose mean and covariance are determined from summary statistics. The Gaussian likelihood assumption allows Markov chain Monte Carlo (MCMC) sampling through a rejection kernel. However, the unbiased estimates of the mean and covariance lead to unbiased posterior estimates; moreover, the MCMC sampler requires the tuning of free parameters. Fearnhead and Prangle [16] proved that the optimal summary statistic for inferring the model parameters, under the assumption of the quadratic loss function, is the true posterior mean given the observed data. They used this fact to model such a posterior mean as a linear model that learns summary statistics directly from data, which is further used in standard ABC. Nonetheless, their semi-automatic approach still has the problem of free parameter selection. Indirect inference, traditionally used in the context of maximum likelihood estimation (MLE), has also been introduced in the context of ABC. Gleim and Pigorsch [26] proposed to use a score vector of an auxiliary model fitted via MLE as summary statistics. Then, comparing the score of the model equipped with the observed data against scores fitted with simulations provides an idea of candidates that follows the posterior distribution. Again, this approach focuses on determining summary statistics rather than an overall automatic ABC.

### 2.2. Weighting-Based Techniques

Comparing summary statistics, to accept or reject candidates depends on thresholds that must be adjusted. An alternative approach introduced by Nakagome et al. [27] stands for a weighting-based inference where all posterior candidates are weighted rather than accepted or rejected. In particular, a conditional mean embedding operator sets up a mapping from summary statistics to model parameters. Such a notion of similarity (assessed in an RKHS) allows setting a probability for the input candidates. While the ABC procedure works in a completely different way, the selection of summary statistics and kernel-free parameters remains. Attempting to avoid the need for summary statistics, Park et al. [20] introduced the maximum mean discrepancy (MMD) criterion to compare probability measures. Using the kernel embedding of distributions into an RKHS, the MMD provides a measure between the distribution of simulated and observed data. It allows assigning a weight to each candidate, using a similarity kernel. The absence of summary statistics comes at the expense of a high computational load. While the authors provided faster approximations for the MMD computation, the need for free parameter selection is not eliminated.

### 2.3. Regression-Adjustment Methods

Different ABC techniques have been proposed for understanding regression as a fundamental concept in machine learning. Mitrovic et al. [28] modeled the functional relationship between simulations and the optimal choice of summary statistics to encode the structure of a generative model. While their flexible framework regulates the kind and amount of information extracted from data, the optimal construction of summary statistics requires multiple sets of free parameters. So, expensive and problem-dependent tuning procedures are yielded. Regarding the number of particles in ABC, Meeds and Welling [29] developed a surrogate model that works as an artificial likelihood to define an adequate amount of simulations in ABC via Gaussian process-based regression. Though the number of required simulations to produce posterior estimations is reduced, the significant difficulty resides in the hyper-parameter tuning. Neural networks and deep learning also have been used to model the relationship between parameter values and summary statistics. Jiang et al. [30] interpreted the posterior mean as a summary statistic by connecting the full dataset to the input layer of a deep neural network (DNN). They attempted to use regularization methods for training the neural network but did not obtain significant improvement. Creel [31] also used DNN to find the posterior mean based on a subset of predefined summary statistics rather than using the full dataset. However, the tuning of a large number of free parameters is still an issue in DNN-based ABC.

## 3. Materials and Methods

In this section, we provide a brief introduction to the ABC fundamentals. First, we introduce the straightforward ABC rejection algorithm and illustrate the usage of kernel methods and Hilbert embedding in the context of ABC. Afterward, we present our automatic ABC approach based on kernel learning and graph theory.

### 3.1. ABC Fundamentals

The central aim of Bayesian statistical inference concerns the calculation of the posterior distribution p(θ|y) for a set of model parameters θ=Θ given the observed data y=X. In particular, the likelihood function p(y|θ) leverages the previous knowledge, as expressed in the prior distribution ζ(θ), into the posterior via Bayes’ theorem. However, when the complexity of the analyzed system leads to an intractable likelihood, neither exact nor sampled posterior p(θ|y)∝p(y|θ)ζ(θ) can be computed. ABC approaches emerged to facilitate such an inference via simulation of the likelihood through a generative model of the system M:Θ→X that is statistically related to a conditional probability p(x|θ), where x=X is a random variable standing for the simulated data [14]. Fundamentally, an ABC-based framework relies on the acceptance and rejection of candidates θ using their corresponding simulated samples *x* based on a distance function $d_{X}:X\times X\to R^{+}$. The rejection in ABC is conducted by sampling multiple model parameters from ζ(θ). The auxiliary model M generates simulations *x* that follow the conditional distribution p(x|θ); then, the subset $\left\{{\theta :d}_{X}\left(x,y\right)<\varepsilon \right\}$ (where $\varepsilon =R^{+}$ is a threshold) is accepted to follow the posterior distribution. In turn, an approximate posterior can be estimated such that:

(1) $$\hat{p}\left(\theta |y;\varepsilon \right)\propto \hat{p}\left(y|\theta ;\varepsilon \right)\zeta \left(\theta \right),$$

where:

(2) $$\hat{p}\left(y|\theta ;\varepsilon \right)=\int_{B\left(y;\varepsilon \right)}p(x|\theta )dx,B\left(y;\varepsilon \right)=\left\{x:d_{X}\left(x,y\right)<\varepsilon \right\}.$$

Notice in Equation (2) how an accurate posterior relies upon an appropriate distance $d_{X}$ and a suitable ε-value. Still, it is often challenging to apply a distance directly on X when dealing with real data since it is commonly formed by a large number of observations and features. In such a case, some alternatives use a mapping s=ϑ(x) before calculating the distance, where s=S is a feature space and ϑ:X→S [17]. The previous setting is widely known as the straightforward ABC rejection algorithm.

**Figure 1:**
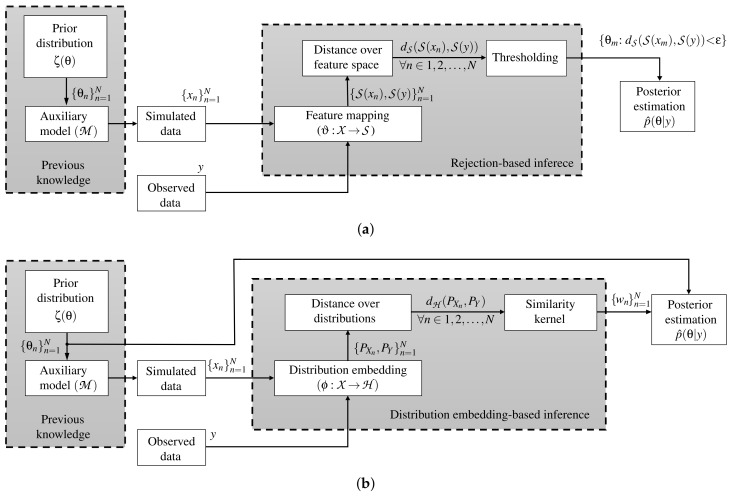
ABC main pipelines. (**a**) ABC rejection algorithm. (**b**) Hilbert embedding-based ABC approach.

Nonetheless, the use of ϑ(x) can lead to a non-sufficient feature space leaking information for complex models. As a consequence, some ABC-based inference approaches approximate the posterior $\hat{p}\left(y|\theta ;\epsilon \right)$ as the convolution of the true likelihood p(y|θ) and a kernel function κ:X×X→R, which imposes a constraint to the rejection of samples as the inner product $\kappa \left(x,y\right)={\langle \phi \left(x\right),\phi \left(y\right)\rangle }_{H}$ in a reproducing kernel Hilbert space (RKHS) H, where ϕ:X→H [20]. In practice, given *N* samples ${\left\{x_{n}∼P_{X_{n}}\right\}}_{n=1}^{N}$ drawn from $p(x|\theta_{n}),$ with $\theta_{n}∼\zeta \left(\theta \right),$ and observed data $y∼P_{Y},$ a weighted sample set $W={\left\{\theta_{n},w_{n}\right\}}_{n=1}^{N}$ is calculated by fixing:

(3) $$w_{n}=\frac{\kappa_{G}\left(d_{H}\left(P_{X_{n}},P_{Y}\right);\epsilon \right)}{\sum_{n=1}^{N}\kappa_{G}\left(d_{H}\left(P_{X_{n}},P_{Y}\right);\epsilon \right)},$$

where $\kappa_{G}\left(d_{H}\left(\cdot ,\cdot \right);\epsilon \right)$ is a Gaussian kernel defined as:

(4) $$\kappa_{G}\left(d_{H}\left(P_{X_{n}},P_{Y}\right);\epsilon \right)=\exp\left(\frac{-d_{H}^{2}\left(P_{X_{n}},P_{Y}\right)}{2\epsilon^{2}}\right),$$

where $\epsilon =R^{+}$ is the kernel bandwidth and $d_{H}:H\times H\to R^{+}$ represents a distance over the Hilbert embedding-based mappings between the distributions $P_{X_{n}}$ and $P_{Y}$. Figure 1 displays the main pipeline of the ABC rejection and Hilbert embedding-based ABC approaches, respectively. Finally, the set *W* is found as in Equation (3) to approximate p(θ|y) via the weighting-based posterior expectation as:

(5) $$\hat{p}\left(\theta |y\right)=\sum_{n=1}^{N}w_{n}\kappa_{G}\left(d_{e}\left(\theta ,\theta_{n}\right);\sigma_{\theta }\right),$$

where $d_{e}\left(\cdot ,\cdot \right)$ stands for the Euclidean distance and $\sigma_{\theta }=R^{+}$ is the kernel width.

### 3.2. Automatic ABC Based on Kernel Learning

**Figure 2:**
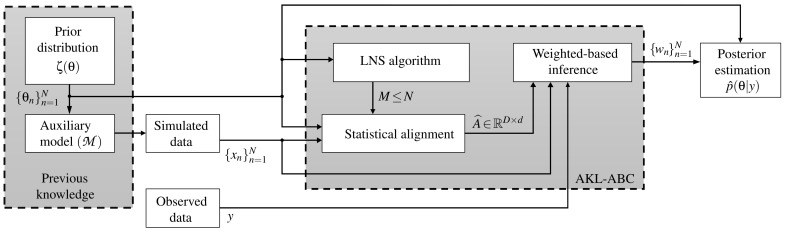
Sketch for the proposed AKL-ABC approach.

Traditionally, the ABC methods do not code the information of the parameter space Θ towards the inference. Namely, the deduction of the model parameters is performed using only explicit information from simulations and observations (see how in Figure 1 there is no direct connection between ${\left\{\theta_{n}\right\}}_{n=1}^{N}$ and the inference stage). Consequently, we introduce a novel kernel learning-based ABC approach, termed automatic kernel learning ABC (AKL-ABC), that codes the similarities between candidates in Θ into the inference stage to obtain an automatic version of ABC. In particular, AKL-ABC comprises a kernel learning stage based on a statistical alignment to assess the matching between similarities defined over parameters and simulations. Moreover, a local neighborhood selection (LNS) algorithm is utilized to highlight local dependencies over candidates in Θ based on graph theory to enhance the kernel similarities through a pruning scheme. The main steps of our AKL-ABC are summarized in the diagram shown in Figure 2 and described below.

#### 3.2.1. Kernel Learning in the Context of ABC

The estimation of the ABC-based posterior as in Equations (3) and (5) requires tuning of the ϵ value. To avoid its tuning, we introduce a statistical alignment approach for enhancing the inference task. The purpose behind this procedure is to include the information contained in the candidates ${\left\{\theta_{n}\right\}}_{n=1}^{N}$ to improve the comparison stage carried out over simulations and observations. Let $\Psi ={\left\{\theta_{n},x_{n}\right\}}_{n=1}^{N}$ be the set of *N* candidates $\theta_{n}=R^{P}∼\zeta \left(\theta \right)$ and their corresponding simulations $x_{n}=R^{Q}∼p\left(x|\theta \right)$. Further, let $\kappa_{\theta }:\Theta \times \Theta \to R^{+}$ be a similarity measure between candidates in Θ that defines the kernel matrix $K_{\theta }=R^{N\times N}$ holding elements:

(6) $$k_{\theta }\left(\theta_{n},\theta_{n^{\prime }}\right)=\left\{\begin{array}{cc} \exp(-d_{\Theta }^{2}\left(\theta_{n},\theta_{n^{\prime }}\right)) & \theta_{n}={Ω}_{n^{\prime }}\\ 0 & \mathrm{otherwise}, \end{array}\right.$$

where ${Ω}_{n^{\prime }}$ is a set holding the *M*-nearest neighbors of $\theta_{n^{\prime }}$ in the sense of the distance $d_{\Theta }:\Theta \times \Theta \to R^{+}$. In this paper, to avoid large variations among components of $\theta_{n}$ we rely on the Mahalanobis distance as follows:

(7) $$d_{\Theta }^{2}\left(\theta_{n},\theta_{n^{\prime }}\right)={\left(\theta_{n}-\theta_{n^{\prime }}\right)}^{⊤}\Sigma_{\Theta }^{-1}\left(\theta_{n}-\theta_{n^{\prime }}\right)$$

where $\Sigma_{\Theta }=R^{P\times P}$ is the sample covariance matrix. Concerning the feature space S, we assess the similarity via the Gaussian kernel function $k_{s}:S\times S\to R^{+}$, $k_{s}\left(\vartheta \left(x_{n}\right),\vartheta \left(x_{n^{\prime }}\right)\right)=\exp\left(-d_{S}^{2}\left(\vartheta \left(x_{n}\right),\vartheta \left(x_{n^{\prime }}\right)\right)/2\gamma^{2}\right)$, to build the matrix $K_{s}=R^{N\times N},$ where $d_{S}^{2}:S\times S\to R^{+}$ and ϑ:X→S is a feature mapping. Here, to perform the pairwise comparison between simulations in S we use also the Mahalanobis distance of the form [32]:

(8) $$d_{S}^{2}\left(\vartheta \left(x_{n}\right),\vartheta \left(x_{n^{\prime }}\right)\right)={\left(\vartheta \left(x_{n}\right)-\vartheta \left(x_{n^{\prime }}\right)\right)}^{⊤}AA^{⊤}\left(\vartheta \left(x_{n}\right)-\vartheta \left(x_{n^{\prime }}\right)\right),$$

where $\Sigma_{S}^{-1}=AA^{⊤}$ stands for the inverse covariance matrix of $\vartheta \left(x_{n}\right)=R^{D}$ and $A=R^{D\times d}$ (d≤D). In this sense, we use the information respecting the similarities among candidates in Θ, represented via $K_{\theta }$, to state a notion of similarity between the simulations and a target observation in S, represented via $K_{s}\left(A,\gamma \right)$. Therefore, we use a CKA-based measure between the kernel matrices as follows [22]:

(9) $$\hat{\rho }\left(K_{\theta },K_{s}\left(A,\gamma \right)\right)=\frac{{\langle {\bar{K}}_{\theta },{\bar{K}}_{s}\rangle }_{F}}{\sqrt{{\langle {\bar{K}}_{\theta },{\bar{K}}_{\theta }\rangle }_{F}{\langle {\bar{K}}_{s},{\bar{K}}_{s}\rangle }_{F}}},$$

where $\bar{K}$ stands for the centered kernel as $\bar{K}=\tilde{I}K\tilde{I}$, being $\tilde{I}=I-1^{⊤}1/N$ the empirical centering matrix, $I=R^{N\times N}$ is the identity matrix, and $1=R^{N}$ is the all-ones vector. Moreover, The notation ${\langle \cdot ,\cdot \rangle }_{F}$ represents the matrix-based Frobenius norm. In Equation (9), $\hat{\rho }\left(\cdot ,\cdot \right)$ is a data-driven estimator that aims to quantify the similarity between the parameter space and the feature space.

To find the projection matrix A, we consider the following optimization problem:

(10) $$\hat{A}=\arg\max_{A,\gamma }\;\log\left(\hat{\rho }\left(K_{\theta },K_{s}\left(A,\gamma \right)\right)\right),$$

where the logarithm function is used for mathematical convenience. Estimation of $\hat{\rho }$ in Equation (10) relies on the explicit objective [33]:

(11) $$\hat{\rho }\left(K_{\theta },K_{s}\left(A,\gamma \right)\right)=\log\left(\mathrm{tr}\left(K_{s}\tilde{I}K_{\theta }\tilde{I}\right)\right)-0.5\log\left(\mathrm{tr}\left(K_{s}\tilde{I}K_{s}\tilde{I}\right)\right)+\rho_{0},$$

where $\rho_{0}=R$ is a constant in A. In this regard, given an initial guess $A^{0}$ (calculated, for instance, using the well-known principal component analysis (PCA) algorithm), the projection matrix A is updated according to the following gradient-descent rule:

(12) $$A^{t+1}=A^{t}-\mu_{A}^{t}\nabla_{A}\left[\hat{\rho }\left(K_{\theta },K_{s}\left(A^{t},\gamma^{t}\right)\right)\right],$$

where $\mu_{A}^{t}=R^{+}$ is the step size of the learning rule and $\nabla_{A}\left[\hat{\rho }\left(\cdot ,\cdot \right)\right]$ stands for the gradient with respect to A of the objective function (11), defined as follows:

(13) $$\nabla_{A}\left[\hat{\rho }\left(K_{\theta },K_{s}\left(A^{t},\gamma^{t}\right)\right)\right]=-4V^{⊤}\left(\Delta \circ K_{s}\right)-\mathrm{diag}\left(1^{⊤}\left(\Delta \circ K_{s}\right)\right)VA^{t},$$

where $V=R^{N\times D}$ is a matrix whose *n*-th row holds the mapped simulation $\vartheta (x_{n})$, and the notations diag(·) and ∘ denote the diagonal operator and the Hadamard product, respectively. Moreover, $\Delta =R^{N\times N}$ is the gradient of the objective function with respect to $K_{s}$:

(14) $$\Delta =\frac{\tilde{I}K_{\theta }\tilde{I}}{\mathrm{tr}\left(K_{s}\tilde{I}K_{\theta }\tilde{I}\right)}-\frac{\tilde{I}K_{s}\tilde{I}}{\mathrm{tr}\left(K_{s}\tilde{I}K_{s}\tilde{I}\right)}.$$

In addition to the optimal learning of the matrix A, the optimization problem in Equation (11) also requires the tuning of the Gaussian kernel bandwidth γ. This joint parameter estimation can be carried out optimizing one variable recursively at a time while the other variable remains unchanged. Namely, the calculation of the rule in Equation (12) is achieved under a constant $\gamma^{t}-$ value. In turn, this kernel bandwidth is estimated (under constant $\hat{A}$) using information theoretic learning (ITL) criteria intending to maximize the overall variability of the information potential ($H_{\alpha }$) computed for all ${\left\{z_{n}=\vartheta {\left(x_{n}\right)}^{⊤}\hat{A}\right\}}_{n=1}^{N}$, so that all the information force magnitudes spread more widely [34]:

(15) $$\gamma^{t}=\arg\max_{\gamma }\mathrm{var}\left\{H_{\alpha }\left(z_{n}|\gamma^{t}\right):n=1,2,\ldots ,N\right\};\alpha =R^{+}.$$

In particular, we use the Renyi’s quadratic entropy, $H_{2}\left(\cdot |\gamma^{t}\right)$, to apply the following gradient-descent update rule over the Gaussian kernel bandwidth:

(16) $$\gamma^{t+1}=\gamma^{t}-\mu_{\gamma }^{t}\nabla_{\gamma }\left[\mathrm{var}\left\{H_{2}\left(z_{n}^{t}|\gamma^{t}\right)\right\}\right],$$

where $\mu_{\gamma }^{t}=R^{+}$ is the step size of the learning rule. See [34] for more details on the derivation of $\nabla_{\gamma }$.

#### 3.2.2. ϵ Tuning Through Nearest Neighbors Based on Graph Theory

Tuning the ϵ-value to approximate the posterior weights as in Equation (3) is a critical step. Depending on the distance output values, a particular choice of ϵ would produce a peaked posterior when just a few numbers of weights have larger values or lead to a posterior similar to the prior distribution in the limit condition when $w_{n}\to 1/N,\forall n=\left\{1,2,\ldots ,N\right\}$. Bearing this in mind, we use the truncated representation of Equation (6) as an alternative to avoid the influence of ϵ via the concept of neighborhood. Namely, an optimal selection of the number of nearest neighbors M=N reveals the prior representative samples in the posterior distribution.

**Figure 3:**
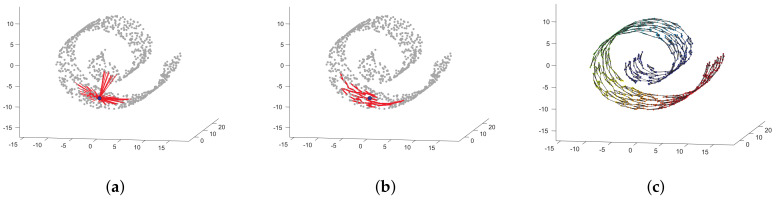
Example of the neighborhoods found by means of Euclidean and geodesic distances. (**a**) Euclidean distance. (**b**) Geodesic distance. (**c**) Completed graph after fixing the number of neighbors using LNS.

The *M*-value can be fixed manually after an exhaustive search based on cross-validation; however, that would hinder the automatic philosophy of this work. To avoid this issue, we use an automatic technique based on locally linear embedding (LLE) and graph theory, the local neighborhood selection (LNS) algorithm, to facilitate the selection of the optimal number of nearest neighbors [35]. LNS aims to identify a suitable number of neighbors for each sample taking into account the structure of the dataset. Specifically, this algorithm is rooted in the idea that when a region around a point is linear and dense, the Euclidean and Geodesic distances obtain a similar set of nearest neighbors for each sample; otherwise, the Euclidean distance will detect short connections while the geodesic distance will identify the right neighbors of each sample. For a better illustration, Figure 3 shows the nearest neighbors for a particular sample in the well-known Swiss-Roll manifold (filled bullet) using both the Euclidean and the geodesic distances. Notice how the Euclidean distance selects neighbors that do not follow the structure of the manifold (Figure 3a) while the geodesic distance understands the actual structure leading to a proper selection of the nearest neighbors (Figure 3b). Figure 3c shows the completed connected graph for all points in the dataset. The LNS algorithm devoted to the estimation of the ABC posterior is described in detail in Appendix A.

Lastly, the ABC-based inference stage is automated via the weighted sample set ${\left\{(\theta_{n},w_{n})\right\}}_{n=1}^{N}$, where each $w_{n}$ is calculated as follows:

(17) $$w_{n}=\frac{\kappa_{E}\left(z,z_{n}\right)}{\sum_{n=1}^{N}\kappa_{E}\left(z,z_{n}\right)},$$

where $\kappa_{E}:R^{d}\times R^{d}\to R$ is a similarity kernel defined as:

(18) $$\kappa_{E}\left(z,z_{n}\right)=\left\{\begin{array}{cc} \exp(-||z-z_{n}|{|}_{2}^{2}) & z_{n}=Z\\ 0, & \mathrm{otherwise}, \end{array}\right.$$

where Z is a set holding the *M*-nearest neighbors of $z=\vartheta {\left(y\right)}^{⊤}\hat{A}$ in the sense of the Euclidean distance.

In short, Algorithm 1 shows the proposed AKL-ABC approach. Furthermore, a MATLAB implementation of the AKL-ABC is publicly available (https://github.com/WilsonGV/AKL-ABC.git).

**Algorithm 1** AKL-ABC algorithm**Input:** Observed data: *y*, prior: ζ(θ), mapping: ϑ.**Output:** Posterior estimation: $\hat{p}\left(\theta |y\right).$
**Kernel learning stage:**
 1:  $\Psi^{{}^{\prime }}={\left\{\left(\theta_{n}^{\prime },x_{n}^{\prime }\right)\right\}}_{n=1}^{N};$$\theta_{n}^{\prime }∼\zeta \left(\theta \right),$$x_{n}^{\prime }∼p\left(x|\theta_{n}^{\prime }\right)$

▹ Draw training data. 2:  M=median{M}▹ Highlight local dependencies in Θ using LNS.
 3:  $\hat{A}=\arg\max_{A}\log\left(\hat{\rho }\left(K_{s}\left(A\right),K_{\theta }\right)\right)$
▹ Compute the CKA based on ϑ, M, and $\Psi^{\prime }$

**Inference stage:**
 4:  $\Psi ={\left\{(\theta_{n},x_{n})\right\}}_{n=1}^{N};$ $\theta_{n}∼\zeta \left(\theta \right),$ $x_{n}∼p\left(x|\theta_{n}\right)$
▹ Draw simulated data. 5:  $z=\vartheta {\left(y\right)}^{T}\hat{A}$▹ Project features of observed data 6:  **for** 
n=1,…,N 
**do**
 7:   $z_{n}=\vartheta {\left(x_{n}\right)}^{T}\hat{A}$▹
▹ Project features of simulated data 8:   ${\tilde{w}}_{n}=\kappa_{E}\left(z,z_{n}\right)$▹ Compute the *n*-th weight value.
 9:  **end for**
 10:  $w_{n}={\tilde{w}}_{n}/\sum_{n=1}^{N}{\tilde{w}}_{n}$▹ Normalize the weights 10:  $\hat{p}\left(\theta |y\right)=\sum_{n=1}^{N}w_{n}\kappa_{G}\left(d_{e}\left(\theta ,\theta_{n}\right);\sigma_{\theta }\right)$
▹ Approximate the posterior.

### 3.3. Theoretical Aspects of AKL-ABC

One of the main contributions of AKL-ABC is the computation of automatic posterior estimates with no need for expensive procedures to select free parameters. We now address two major theoretical aspects of AKL-ABC: Learning performance and computational complexity.

To investigate the learning performance of AKL-ABC, we must analyze the CKA-based measure over matrices $K_{s}=R^{N\times N}$ and $K_{\theta }=R^{N\times N}$. The empirical alignment $\hat{\rho }\left(K_{s},K_{\theta }\right)$, as defined in Equation (9), is a statistical approximation of the true alignment $\rho (k_{s},k_{\theta })$ defined as [36]:

(19) $$\rho \left(k_{s},k_{\theta }\right)=\frac{{\langle k_{s},k_{\theta }\rangle }_{P}}{{\langle k_{s},k_{s}\rangle }_{P}{\langle k_{\theta },k_{\theta }\rangle }_{P}},$$

where ${\langle f,g\rangle }_{P}=\int f\left(a,b\right)g\left(a,b\right)dP\left(a\right)dP\left(b\right)$ based on the input distribution P.

**Theorem** **1.**  *(Cortes et al.* [22]) *Assume $k_{s}\left(\cdot ,\cdot \right)\leq \varpi$ and $k_{\theta }\left(\cdot ,\cdot \right)\leq \varpi^{\prime }$. For any ς>0, with probability at least 1−ς, the following inequality holds:*

$$|\rho \left(k_{s},k_{\theta }\right)-\hat{\rho }\left(K_{s},K_{\theta }\right)|\leq 18\Lambda \left[\frac{3}{N}+8\sqrt{\frac{\log\frac{6}{\varsigma }}{2N}}\;\right],$$

*where $\Lambda =\max(\varpi \varpi^{\prime }/E\left[k_{s}^{2}\right],\varpi \varpi^{\prime }/E\left[k_{\theta }^{2}\right])$.*

**Proposition** **1.** *Given the sample set ${\left\{(\theta_{n},w_{n})\right\}}_{n=1}^{N}$, where the weights $w_{n}$ are fixed according to the AKL-ABC algorithm, the expectation $E\left[\hat{p}\left(\theta |y\right)\right]=\sum_{n=1}^{N}w_{n}\theta_{n}$ tends to the expected value of the posterior distribution as N→∞.*

**Proof.**  According to Theorem 1, the empirical CKA-based alignment used in AKL-ABC ($\hat{\rho }\left(K_{s},K_{\theta }\right)$) tends asymptotically to the true statistical alignment ($\rho (k_{s},k_{\theta })$) as the number of input points tend to infinity. As a consequence, $K_{s}-K_{\theta }$ tends to the null matrix as N→∞ meaning that the notion of similarity defined over simulations and parameters spaces is the same. Then, the neighborhood around projected features of observed data $z=\vartheta {\left(y\right)}^{⊤}\hat{A}$, according to Equations (17) and (18), leads to a neighborhood around the true value of the model parameters in the sense of the statistical input distribution P. □

**Remark** **1.** *While the LNS algorithm operates over the input set ${\left\{\theta_{n}\right\}}_{n=1}^{N}$ (see Appendix A), the concept of neighborhood is directly transferred from the parameter space to the simulations space. Namely, the similarity assessment is exchangeable from one space to another due to the asymptotic behavior of the statistical alignment with the number of input points.*

Finally, taking into account the main steps of AKL-ABC, we can find its computational complexity: The LNS algorithm requires $O(PN^{2}+\varrho N^{2})$ operations, where $\varrho \leq \lceil \sqrt{N}\rceil$ takes a dynamic integer value since the LNS method determines the number of neighbors by adapting the dataset distribution (see Appendix A) [35]; furthermore, a number of $O(N^{2}+GN^{2})$ operations is required to find the projection matrix $\hat{A}$ based on $K_{s}$ and $K_{\theta }$, where *G* is the number of desired gradient-descend steps. Then, the total complexity of our AKL-ABC is $O(N^{2}\left(P+\varrho +G+1\right))$.

## 4. Experimental Setup

To evaluate the performance of our AKL-ABC approach, we analyze three different attributes: (i) Posterior quality approximation in applications that comprise both synthetic and real datasets, (ii) suitable convergence concerning the number of ABC samples (*N*), and (iii) adequate selection of the number of nearest neighbors (*M*).

### 4.1. Datasets and Quality Assessment

We examine the next experiments following [20]: A toy problem concerning synthetic data from a mixture model and a Bayesian inference problem for a real-world ecological dynamic system. Each experiment is described below.

Toy problem: We inspect a mixture of uniform distributions of the form:

(20) $$p\left(x|\pi \right)=\sum_{c=1}^{C}\pi_{c}U(c-1,c),$$

where $\pi ={\left\{\pi_{c}=R^{+}\right\}}_{c=1}^{C}$ stores the mixing coefficients holding $\sum_{c=1}^{C}\pi_{c}=1$, and C=N is the number of components. Here, the aim is to estimate the posterior p(π|y) for C=5, given synthetic observations *y* drawn from the mixture with true parameters (target): $\pi^{*}=\left\{0.25,0.04,0.33,0.04,0.34\right\}$. Besides, as quality assessment for the toy problem we utilize the Euclidean distance as follows [20]:

(21) $$E=\left|\right|\pi^{*}-\hat{\pi }{\left|\right|}_{2},$$

where $\hat{\pi }$ is the expected value of the posterior found using the weights ${\left\{w_{n}\right\}}_{n=1}^{N}$.

Real-world problem: Inference tasks over dynamic ecological systems representing chaotic and near-chaotic domains is quite a challenge [7,9]. We considered the problem of inferring the dynamics of an adult blowfly population. Particularly, Wood [18] introduced a model for describing population dynamics using a differential equation as follows:

(22) $$N_{t+1}=PN_{t-\tau }\exp\left(-\frac{N_{t-\tau }}{N_{0}}\right)e_{t}+N_{t}\exp\left(-\delta \epsilon_{t}\right),$$

where $N_{t+1}$ denotes the observation time at t+1, which is determined by the time-lagged observations $N_{t}$ and $N_{t-\tau }$. Moreover, $e_{t}$ and $\epsilon_{t}$ stand for the Gamma distributed noise as $e_{t}∼G\left(1/\sigma_{p}^{2},\sigma_{p}^{2}\right)$ and $\epsilon_{t}∼G\left(1/\sigma_{d}^{2},\sigma_{d}^{2}\right)$, respectively. Here, our aim is to estimate the posterior of the parameters $\theta =\{P,N_{0},\sigma_{d},\sigma_{p},\tau ,\delta \}$ given observed data concerning a time series of 180 observations (available in the Supplementary Materials of [18]). For concrete testing, we adopt log-normal distributions for setting priors over θ[29]: $\log\left(P\right)∼N\left(2,2^{2}\right),$$\log\left(N_{0}\right)∼N\left(6,1\right),$$\log\left(\sigma_{d}\right)∼N\left(-0.5,1\right),$$\log\left(\sigma_{p}\right)∼N\left(-0.5,1\right),$log(τ)∼N(2.7,1),$\log\left(\delta \right)∼N\left(-1,0.4^{2}\right)$.

On the other hand, because in a real-world inference task there is no target value for the model parameters, the quantitative assessment must rely on the quality of predictions. Thus, the Euclidean distance is computed to measure the performance in the feature space as follows [20]:

(23) $$E=||\vartheta \left(y\right)-\vartheta \left(x_{n}|\hat{\theta }\right){\left|\right|}_{2},$$

where $x_{n}|\hat{\theta }$ is a simulation from the model given the expected value of the posterior.

### 4.2. AKL-ABC Training and Method Comparison

For comparison purposes, we considered the K2-ABC approach proposed by Park et al. [20] due to its excellent performance over other methods such as: Indirect score ABC (IS-ABC) [19], semi-automatic ABC (SA-ABC) [16], kernel ABC (K-ABC) [27], and synthetic likelihood ABC (SL-ABC) [18]. We selected the previous benchmark since they belong to the two main groups of ABC algorithms considered in the scope of this paper, the ones that compute posterior estimations based on summary statistics (IS-ABC, SA-ABC, SL-ABC), and the ones that produce posterior estimates via weighting-based inferences (K-ABC, K2-ABC). In particular, K2-ABC uses a mapping of the observed and simulated data from the simulation space to an RKHS and employs a maximum mean discrepancy (MMD) criterion to construct a dissimilarity measure between distributions of observed and simulated data. While this method has outstanding results, it requires the tuning of free parameters. On the other hand, we can find the best possible performance of our AKL-ABC by running the inference stage in Algorithm 1 with ${\tilde{w}}_{n}=\kappa_{E}\left(\pi^{*},\pi_{n}\right)$; we refer to this approach as “best”.

Regarding the toy problem, we draw N=1000 samples from a symmetric Dirichlet prior, π∼Dirichlet(1), and then used the mixture model to form the simulated data by drawing 400 observations for each prior candidate. Moreover, we employ a histogram with ten bins as feature mapping (ϑ) in AKL-ABC and fix the Gaussian kernel bandwidths as 0.1 and 0.001 for the characteristic and similarity kernels, respectively, in the K2-ABC method. These parameters were tuned according to the cross-validation procedure planted by Park et al. [20].

For the real-world problem, we generate N=5000 samples from the prior and then assess the model to form the simulated data by drawing 180 observations for each prior candidate. Besides, as feature mapping (ϑ), we selected the ten statistics used by Park et al. [20]. Furthermore, due to fluctuations produced by $\epsilon_{t}$ and $e_{t}$, we draw 100 simulations from the model given the expected value of the posterior and compute the boxplots of E for each method.

As suggested by authors in [32], we fixed the free parameters of the CKA algorithm regarding the gradient-descend optimization as follows: The adaptive step size of the learning rules are adjusted such that $\mu_{\gamma }^{t}$ and $\mu_{A}^{t}$ decrease gradually from 1e−4 to 1e−5 through a maximum number of iterations empirically limited up to 100. Moreover, the initial guess for the rotation matrix $A^{0}$ is computed using the well-known PCA method retaining the 95% of the variance explained (which defines the number of columns of A, d≤D), while $\gamma^{0}$ is calculated as the median of the input data Euclidean distances.

## 5. Results and Discussion

### 5.1. Toy Problem Results

**Figure 4:**
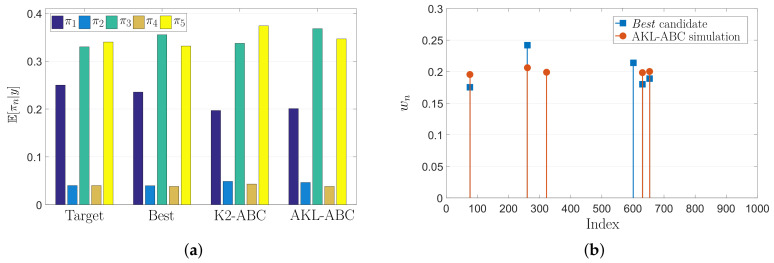
Uniform mixture model results. (**a**) Estimated mean posterior of mixing coefficients using various methods. (**b**) Weights of the first five nearest neighbors in AKL-ABC.

Since this is a full controlled experiment with known parameters $\pi^{*},$ we can find the best possible performance of our AKL-ABC by running the inference stage with ${\tilde{w}}_{n}=\kappa_{E}\left(\pi^{*},\pi_{n}\right)$. The previous setting is equivalent to think that the statistical alignment found via CKA between $K_{\theta }$ and $K_{s}$ yields to $K_{\theta }$ = $K_{s}$. Figure 4 shows the “best” performance along with K2-ABC and AKL-ABC results over the uniform mixture problem. In Figure 4a, the expected value of the posterior computed for all methods is close to the target. In particular, we obtained $E_{Best}=0.030\pm 0.039$, $E_{K2-\mathrm{ABC}}=0.063\pm 0.042$, and $E_{\mathrm{AKL}-\mathrm{ABC}}=0.064\pm 0.041$. Notice how the “best” approach achieves the lowest possible error providing a lower bound that the AKL-ABC could reach in the ideal case of perfect statistical alignment. These results show that our AKL-ABC is a competitive estimator to K2-ABC with a significant advantage concerning the automatic selection of free parameters without requiring any tuning procedure. Besides, to provide a better understanding of the AKL-ABC effectiveness, in Figure 4b we show the weights for the five nearest neighbors (according to the LNS algorithm) used to compute the posteriors. As noted, the majority of the chosen simulations for AKL-ABC match the selected candidates using the “best”, although our approach never observes the target.

**Figure 5:**
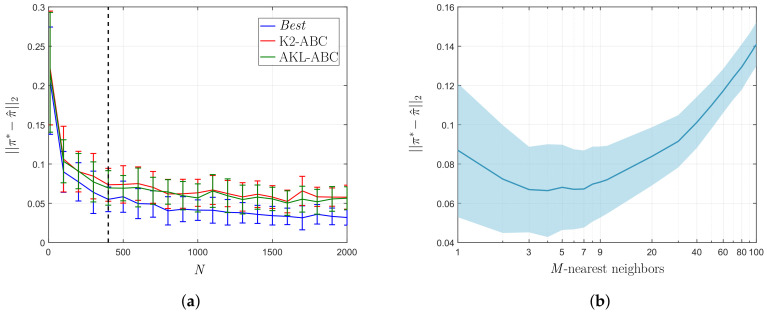
Posterior approximation error over the uniform mixture model. (**a**) E vs. *N* curve. (**b**) E vs. *M* curve.

To observe the stability of the proposed AKL-ABC concerning the number of simulations, we select $y^{*}$ to be a set of 400 synthetic observations drawn from the model (see Equation (20)) using the target $\pi^{*}$. Then, we solve repeatedly the problem of inferring an approximation for $p(\theta |y^{*})$ by using each method, increasing *N* with a step-size of 100. Figure 5a shows the resulting E vs. *N* curve where the larger the number of simulations the lower and more confident the approximation error. While our AKL-ABC approach obtains a similar performance in comparison with the K2-ABC method in terms of stability and approximation error, our methodology overcomes the benchmark regarding its automatic philosophy that avoids any tuning procedure.

In turn, we compare the performance of the LNS algorithm concerning the choice of the *M*-nearest neighbors in AKL-ABC against a conventional grid search using $y^{*}$ as observed data and *N* = 400 simulations. The E vs. *M* curve in Figure 5b proves that either small or large neighborhoods result in significant errors because, in the first case, the data structure is weakly encoded and, in the second case, the estimation is biased towards the population average. Besides, the larger the number of neighbors, the more confident the posterior estimate, since the set of weights ${\left\{w_{n}\right\}}_{n=1}^{N}$ contains a larger number of non-zeros values. Therefore, introducing the LNS algorithm into the AKL-ABC results in an automatically computed number of neighbors (*M* = 5) lying on the minimum error region.

### 5.2. Real Dataset Results

**Figure 6:**
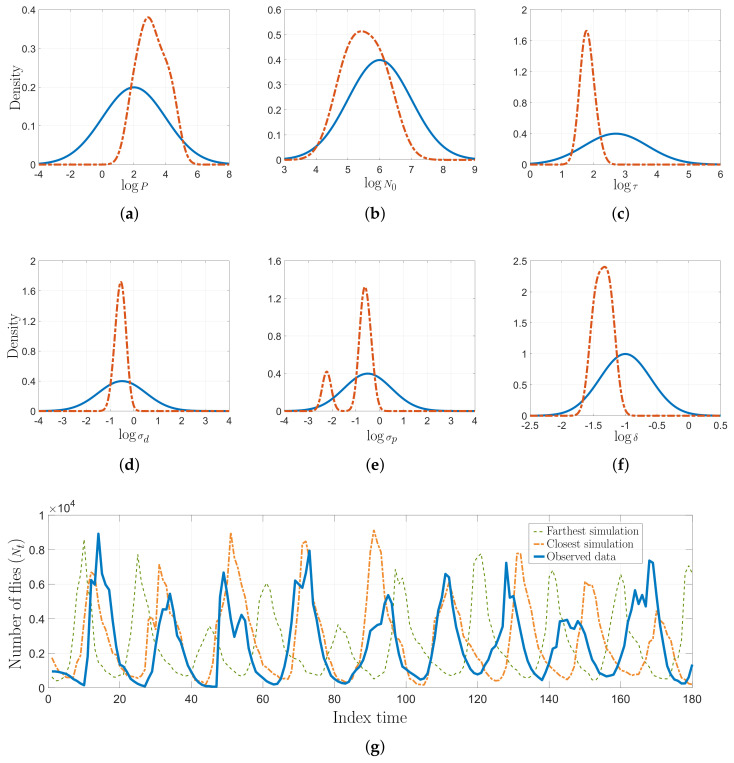
Non-linear ecological dynamic system results. (**a**–**f**) Prior distribution (solid line) and AKL-ABC-based posterior estimation (dashed line) of model parameters in the log-space. (**g**) Some predictions from the model using the expected value of the parameters found via AKL-ABC.

**Figure 7:**
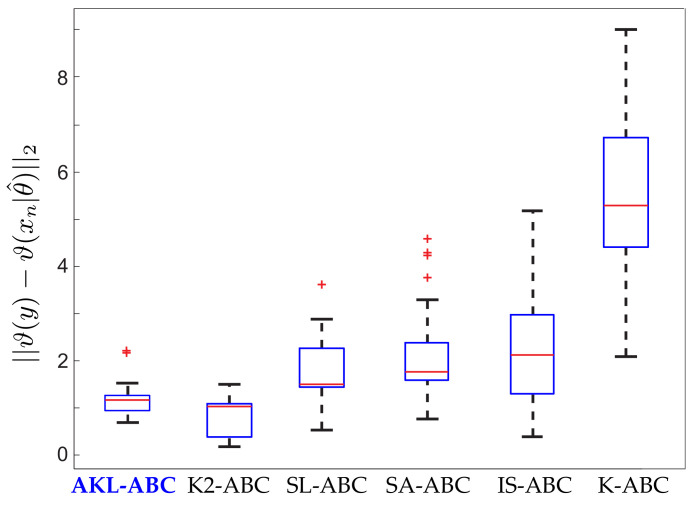
Performance of our AKL-ABC against the different ABC methods tested over the blowfly dataset by Park et al. [20].

Inferring the model parameters in this blowfly dataset is a very challenging task since the system dynamics can easily move from stable to chaotic regimes. The auxiliary model would produce completely different simulations in face of minimal fluctuations of the parameters [18,29]. This states an interesting scenario to test the performance and robustness of our AKL-ABC. In Figure 6, we provide the prior and the posterior approximations for each parameter, fixing $\sigma_{\theta }$ according to [37]. Notice how our proposal updates the beliefs about the model parameters leading to more concentrated posteriors. In the case of $\log(\sigma_{p})$, two modes reflect different intervals with probable values for driving the noise realization associated with egg production in the blowfly population. However, there is a predominant mode that states higher probabilities for this parameter. Moreover, Figure 6g shows the closest and farthest predictions to the observed data selected from 100 realizations used to compute E, showing that the inferred posterior lays on a stable regime. Finally, Figure 7 shows the performance of AKL-ABC compared against different ABC-based methods tested on the blowfly dataset by Park et al. [20]. As seen, our proposed method is a quite competitive approach to the benchmark. In particular, the confidence intervals of E are (1.0620,1.1232), (0.9923,1.1543), and (1.8401,2.0464) for the AKL-ABC, K2-ABC, and SL-ABC methods, respectively. The smaller the confidence interval, the more stable the mean posterior prediction since the model dynamic straightforwardly falls into chaotic regimes, even with minimal changes of the model parameters. Thus, the narrowest confidence interval obtained in AKL-ABC proves its capability to deal with complex dynamic data. Furthermore, AKL-ABC holds a significant advantage concerning the automatic selection of free parameters.

### 5.3. Computational Tractability

One of the primary considerations in ABC is computational tractability. Because there are no tuning procedures required in our AKL-ABC, the time complexity for a given dataset ${\left\{\theta_{n}=R^{P},\vartheta \left(x_{n}\right)=R^{D}\right\}}_{n=1}^{N}$ is $O(N^{2}\left(P+\varrho +G+1\right))$. Nonetheless, in the case of other non-automatic ABC methods, the number of free parameters and the grid they define notably augment the computational burden. Namely, if the number of required operations for a given ABC method is denoted by O(N), the final time complexity for tuning *F* free parameters is $O(N\left(\prod_{i=1}^{F}\lambda_{i}\right))$, where $\lambda_{i}=N$ is the number of all possible values to be explored for the *i*-th free parameter. Notice that the thinner the exploration grid, the more prohibited the number of overall operations. For instance, the K2-ABC approach requires $O(N^{2})$ operations [20]. However, the grid search needed for tuning the free parameters increases the computational complexity exponentially to $O(N^{2}\left(\lambda_{1}\lambda_{2}\right))$, where $\lambda_{1},\lambda_{2}$ are related to grids defined over characteristic and similarity kernel widths. See that the performance of AKL-ABC depends on ϱ and *G*; however, in practice, the CKA and LNS algorithm have a fast convergence [32,35].

## 6. Conclusions

In this paper, we focus on the problem of automatically performing Bayesian statistical inference under the intractability of the likelihood function. In particular, we propose an automatic enhancement of the well-known ABC algorithm devoted to approximate Bayesian inference called AKL-ABC. In particular, we include a metric learning approach based on a CKA framework to quantify the statistical alignment between parameter and simulation spaces in ABC. Then, a Mahalanobis distance is learned through CKA, and a graph representation based on a local neighborhood selection algorithm is employed to reveal local relationships among parameter and simulation samples. Notably, AKL-ABC has an advantage over other ABC approaches: The statistical alignment over parameter and simulations spaces and the concept of neighborhood introduce additional information in the inference procedure such that the overall ABC framework does not require the tuning of any free parameters. Attained results on a synthetic dataset and a real-world ecological system show the introduced AKL-ABC is robust to substantial changes in data dynamics and produces quite competitive posterior approximation compared to other non-automatic state-of-the-art ABC methods.

Future work includes the extension of AKL-ABC for high-dimensional problems where a large number of observations could be prohibited, via a possibly global neighborhood selection approach that supports a faster computation of the number of neighbors (*M*) required in AKL-ABC, taking into account comprehensive features rather than local properties of the input data. Moreover, the inclusion of other dissimilarity measures besides the Mahalanobis distance, coupled with the neighborhood-based philosophy of AKL-ABC, is also a potential line of research to deal with applications that gather complex and noisy data. Lastly, the computational burden would be enhanced based on stochastic gradient approaches [38].

## Abbreviations

**Symbol**	**Description**
Θ	Parameter space
X	Simulations space
S	Feature space
H	Reproducing Kernel Hilbert Space (RKHS)
$\theta_{n}$	*n*-th prior sample
$x_{n}$	*n*-th simulation
*y*	Observed data
$w_{n}$	Representation weight associated to the *n*-th prior sample
$z_{n}$	Projection of the *n*-th simulation
${Ω}_{n}$	Set of the *M*-nearest neighbors of $\theta_{n}$ according to the LNS algorithm
Z	Set of the *M*-nearest neighbors of projected features of the observed data
M	Auxiliary model
ϑ	Feature mapping
ϕ	Mapping function associated to the RHKS H
κ(·,·)	Kernel function
$\kappa_{G}$	Gaussian kernel
$\kappa_{E}$	Similarity kernel
$\hat{\rho }\left(\cdot ,\cdot \right)$	Statistical alignment between two kernel matrices
$H_{\alpha }$	α-order Information Potencial
$d_{\Theta }$	Distance between prior samples
$d_{X}$	Distance between simulations
$d_{S}$	Distance between features of simulations
$d_{H}$	Distance between distributions in an RKHS
$d_{e}$	Euclidean distance
ζ(θ)	Prior distribution
p(θ|y)	Posterior distribution
p(y|θ)	Likelihood function
p(x|θ)	Conditional distribution associated to simulations
$P_{X_{n}}$	Distribution of the *n*-th simulation
$P_{Y}$	Distribution of the observed data
$K_{\theta }$	Kernel matrix over prior samples
$K_{S}$	Kernel matrix over features of simulations
A	Projection matrix for the Centered Kernel Alignment (CKA)
$\Sigma_{\Theta }$	Sample covariance matrix of prior samples
V	Feature matrix of simulations
ε	Threshold for rejection ABC
$\epsilon ,\gamma ,\sigma_{\theta }$	Gaussian kernel bandwidths
*M*	*M*-nearest neighbors according to the LNS algorithm
ϱ	number of iterations performed by the LNS algorithm
*G*	number of iterations required by the gradient-descent method in AKL-ABC
$\mu_{A},\mu_{\gamma }$	Step sizes for gradient descent rules

## Appendix A. Local Neighborhood Selection (LNS) Algorithm

Given an input dataset $\Theta ={\left\{\theta_{n}\right\}}_{n=1}^{N}$, the following are the main steps of the LNS algorithm [35]:

1. Compute the Euclidean distance $d_{e}$ for all points in Θ.
2. Construct the minimal connected neighborhood graph G of the given dataset Θ by the *M*-nearest neighbors method (MNN) fixing the smallest neighborhood size $m_{min}=1$. Check the full connectivity of the graph by using the Breadth-first search (BFS) [39]. If the graph is not full connected, update $m_{min}=m_{min}+1$ and start again this step.
3. Compute the geodesic distance $d_{g}$ over G by using the Dijistra’s algorithm [39].
4. Define $m_{max}=N^{2}/\left(m_{min}N_{G}\right)$, where $N_{G}$ is the number of edges in G and *N* the number of samples in Θ.
5. Set the vector $M_{i}=\left[m_{min}+1,\ldots ,m_{max}\right]$, with $M_{i}=R^{B}$. The vector $M_{i}$ contains the possible values of *m* for each $\theta_{i}$.
6. For each $\theta_{i}$ define the sets $T_{d_{e}}^{\left(b\right)}$ and $T_{d_{g}}^{\left(b\right)}$, with b={1,…,B}. Each element in $T_{d_{e}}^{\left(b\right)}$ and $T_{d_{g}}^{\left(b\right)}$ corresponds to the $m_{i_{b}}$ nearest neighbors $\theta_{j}$ of $\theta_{i}$ ($j=\{1,\ldots ,m_{i_{b}}\}$) according to the Euclidean and geodesic distances, respectively.
7. Calculate the linearity conservation matrix $L=R^{N\times B}$, which analyses the similarity of the neighborhoods obtained by $d_{e}$ and $d_{g}$, taking into account the patch size. Each element of L can be computed as, $\ell_{ib}=\left|\left\{\bar{T_{d_{e}}^{\left(b\right)}\cap T_{d_{g}}^{\left(b\right)}}\right\}\right|/m_{i_{b}}$, where $\left|\cdot \right|$ calculates the cardinality of a set and $\bar{\left\{\cdot \right\}}$ the complement.
8. Initially, for each $\theta_{i}$ define the set $M_{o}=\emptyset$. Verify the equality $\ell_{ib}=\min\left\{V_{i}\right\}$, where $V_{i}=R^{B}$ is the *i*-th row vector of L. If the equality is fulfilled, then update $M_{o}=M_{o}\cup m_{i_{b}}$.
9. Define $m_{i}$ for each $\theta_{i}$ as $m_{i}=\max\left\{M_{o}\right\}$.
10. Smooth $m_{i}$ to obtain similar properties in surrounding neighborhoods according to $m_{i}=\left(m_{i}+M_{O}1\right)/\left(m_{i}+1\right)$, where $M_{O}=R^{m_{i}}$ is a vector with the sizes of the neighborhoods of each element in O (set with the first $m_{i}$ nearest neighbors $\theta_{j}$ of $\theta_{i}$ according to the Euclidean distance, $j=\{1,\ldots ,m_{i}\}$).
11. Store all the values $m_{i}$ into the vector M.
12. Remove the outliers in M (see [40]), and replace them by the average of the elements in M, which were not identified as outliers.
13. Each element in M contains the number of nearest neighbors $m_{i}$ for each $\theta_{i}$.

Subsequently, a global representation of the manifold is accomplished by defining the *M*-value as M=median{M} to avoid possible irregular neighborhood sizes.
